# Employment as a Cause of Scrofula

**Published:** 1846-09

**Authors:** 


*Employment as a Cause of Scrofula.*—In considering this question, Mr. Phillips devotes his principal attention to the litigated question of the influence of factory labor in developing scrofula. He has secured several valuable and extensive statistical returns from the manufacturing districts; the substance of which we subjoin in his own words:

"In Leeds, Dr. T. Smith very kindly examined, at my request, 1095 children, employed in different factories, and examined 548 children of the same class, not employed in factories. The result is, that those not employed in factories exhibited marks of scrofula in 8 per cent. more instances than those whose days are spent in such establishments. In Manchester and other great manufacturing towns, a similar result has been obtained by examination made at my request, and on so large a scale that there is every reason to feel confident in the opinion already expressed. Again, from his dispensary practice, Dr. Smith has made me the following report:—Of

916 persons, between 7 and 14, the children of factory operatives, but not themselves employed in factories, 365, or 39 per cent., had enlarged glands, and 75, or 8 per cent., had scars resulting from scrofula. Of 567 persons, all under 21, and employed in factories, 124, or 22 per cent., had scrofulous scars."

"Mr. Poyser, the intelligent surgeon of Winksworth, kindly examined for me the people employed in Mr. Arkwright's mills, and the following are the results which he communicated:—'persons examined, 798; total number having marks of scrofula, 29.'

· · · · · "I have obtained, through the kindness of Messrs. Horner and Saunders, the results of the examination of 6754 factory children, from which it appears that marks of scrofula were found in only 905 instances, or only 13½ per cent. The returns of Mr. Freday, Mr. Deavis, and that of other friends, who have kindly made a comparative examination of a large number of children, exhibit similar results; and they leave no doubt on my mind that children employed in factories are more free from scrofula than the average of children in England and Wales."—*Brit. and For. Med. Rev.*

